# Influences of Sedimentary Environments and Volcanic Sources on Diagenetic Alteration of Volcanic Tuffs in South China

**DOI:** 10.1038/s41598-018-26044-w

**Published:** 2018-05-16

**Authors:** Nina Gong, Hanlie Hong, Warren D. Huff, Qian Fang, Christopher J. Bae, Chaowen Wang, Ke Yin, Shuling Chen

**Affiliations:** 1 State Key Laboratory of Biogeology and Environmental Geology, School of Earth Sciences, China University of Geosciences, Wuhan, 430074 China; 2 Gemological Institute, China University of Geosciences, Wuhan, 430074 China; 3 Department of Geology, University of Cincinnati, Cincinnati, OH 45221-0013 USA; 4 Department of Anthropology, University of Hawaii at Manoa, 2424 Maile Way, 346 Saunders Hall, Honolulu, HI 96822 USA

## Abstract

Permian-Triassic (P-Tr) altered volcanic ashes (tuffs) are widely distributed within the P-Tr boundary successions in South China. Volcanic altered ashes from terrestrial section-Chahe (CH) and marine section-Shangsi (SS) are selected to further understand the influence of sedimentary environments and volcanic sources on diagenetic alterarion on volcanic tuffs. The zircon ^206^Pb/^238^U ages of the corresponding beds between two sections are almost synchronous. Sedimentary environment of the altered tuffs was characterized by a low pH and did not experience a hydrothermal process. The dominant clay minerals of all the tuff beds are illite-smectite (I-S) minerals, with minor chlorite and kaolinite. I-S minerals of CH (R3) are more ordered than SS (R1), suggesting that CH also shows a higher diagenetic grade and more intensive chemical weathering. Besides, the nature of the volcanism of the tuff beds studied is derived from different magma sources. The clay mineral compositions of tuffs have little relation with the types of source volcanism and the depositional environments. Instead, the degree of the mixed-layer clay minerals and the REE distribution are mainly dependent upon the sedimentary environments. Thus, the mixed-layer clay minerals ratio and their geochemical index can be used as the paleoenvironmental indicator.

## Introduction

The Permian-Triassic boundary (PTB) mass extinction event was the most profound biotic crisis in the Phanerozoic, severely affecting most land and marine taxa^1–3^. However, the trigger that prompted the PTB mass extinction event continues to be intensely debated and has yet to reach a consensus^4^. The most popular hypothesis is that contemporaneous extensive volcanic activities initiated the mass extinction^1,5–8^, while other triggers that have been proposed include oceanic anoxia, bolide impact and sea-level change^9^. The geographically widespread altered tuffs, especially those that are potassium enriched, can also be called K-bentonites. The tuffs represent significant stratigraphic markers for regional correlation, permitting geochemical characterization of the parent magmas and tectonic background of the source volcanism^10,11^.

In south China, the PTB ash beds are well-exposed and continuous with relatively significant diagenetic and metamorphic overprints^12,13^. The PTB ash beds have been frequently studied, primarily for their paleontological significance, REE and Hf-isotope analyses of zircons, and for clay mineralogical investigations^2,14–16^. However, there are very few studies focused on the diagenetic influences of different depositional environments based on mineralogy and geochemistry of the tuffs and the details of clay minerals. As clay minerals are sensitive indicators of their depositional environments, analyses of the volcanic altered ashes are necessary to further understand the role of tuff beds^17^. Hong *et al*. (2017) studied the littoral and interactive marine-terrestrial sections’ PTB ashes in Guizhou Province and suggested that a stratigraphic correlation can be identified from the geochemical fingerprinting of the ash beds.

Volcanic ashes are usually preserved in aqueous environments and further altered into several types of clay minerals^18^. The transformation process is relatively rapid, even within several days to a few years, with the *in-situ* migration of major and trace elements^18–21^. The formation of clay minerals within volcanic ash beds is influenced by the parent rock, sedimentary environment, interaction of the glass with water, pH, water/rock ratios, and the involvement of organic matter^17,22–24^. Volcanic activities during the P-Tr transition were continuous, large-scale and isochronous^6,10,25^. As the tuff beds exist widely during the P-Tr transition and are well-preserved in South China^14,16,26^, their detailed investigations are significant to obtain a better understanding of the correlations between the source magmas, sedimentary environments, altered clay minerals, and diagenetic processses. In order to explore the correlation of PTB volcanic altered ashes in different facies, this study concerns marine and terrestrial successions in the lower Yangtze Regions, and focuses on the environmental impact on the tuff alteration, the function of scale chronostratigraphic markers, and the possible origin of the tuffs.

## Geological background

**Figure 1:**
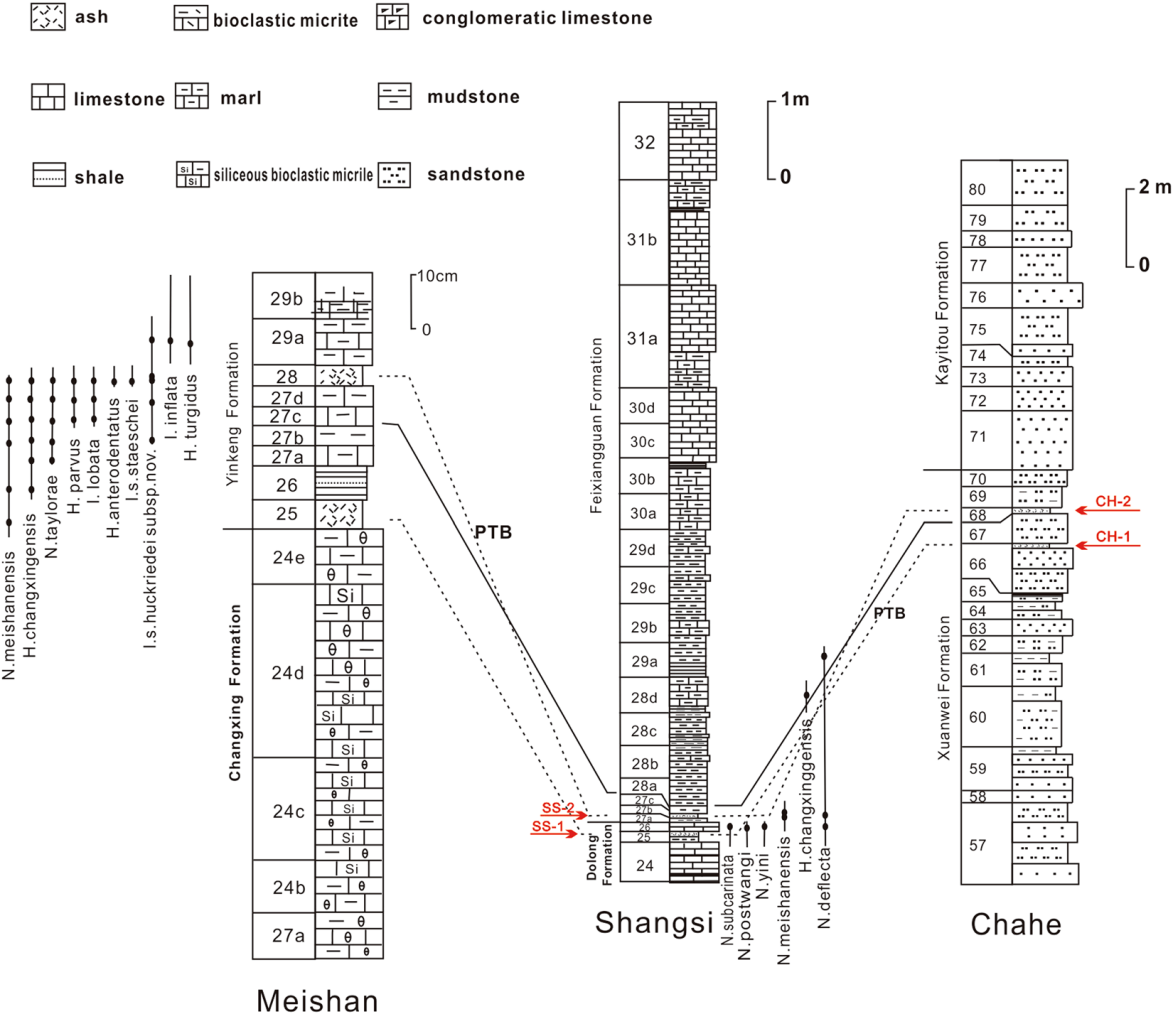
Lithological columns of the correlation and sampling horizons of the studied sections. Meishan section is the standard P-T profile. Red arrows point out the corresponding studied beds. Lithological columns of Meishan and Shangsi are referenced from Jiang (2008).

The studied sections from the Lower Yangtze Block were located in the eastern part of the Palaeo-Tethys Ocean during the P-Tr transition, which connected the Qinling paleo-ocean and the Cathaysian paleo-block^15^. The studied terrestrial section is situated at Chahe (CH), Heishitou town in western Guizhou Province, and is located in the upper part of the Xuanwei Formation. The Permian Xuanwei Formation is comprised of fluvial and lacustrine units, and the change of the formation is from meandering fluvial to lacustrine (Beds 56–80) for a deepening and transgressive process^2^. According to the previous stratigraphic studies^27^, layers 68–70 are the Permian-Triassic Transition Beds (PTTB). Two typical K-bentonite layers, bed 66 f (10 cm) and 68a (8 cm), are interbedded with coal measures and yield abundant Permian terrestrial fossil plants, β-quartz, apatite and zircon. Bed 68 has been identified as the PTB, on the basis of zircon SHRIMP technique zircon ^206^Pb/^238^U date of 252.3 ± 0.07 Ma, correlating with bed 25 at the Meishan section^2,28^. Beds 66 f and 68a closest to the PTB were chosen as the samples and numbered CH-1 and CH-2 (Fig. 1).

The marine Shangsi (SS) section consists of the Dalong Formation and Feixianguan Formation, located at Shangsi town, Sichuan Province, South China. The SS section preserves a continuous depositional environment from an upper slope sedimentary environment in the Late Permian to a tidal flat environment in the early Triassic^29,30^. Beds 23, 25, 27 and 28 are intercalated with K-bentonites presumed to be volcanogenic in origin^31^. The PTB has tentatively been placed on the top of bed 27 on the basis of zircon U-Pb age, 252 ± 0.13 Ma, presented by Shen *et al*.^27^. Besides, according to the conodont stratigraphy, the PTB was placed ~22 cm above the bottom of bed 28^30^ correlating with the GSSP Meishan Bed 25^32^. Based on the PTB studied before, beds 25 and 28 were selected in this study and numbered SS-1 and SS-2 (Fig. 1). The bed 27 are primarily composed of pale yellow K-bentonites, and the bed 28 are mainly dolomitic mudstones intercalated with the K-bentonites^27^.

## Results

### Zircon U-Pb ages

**Figure 2:**
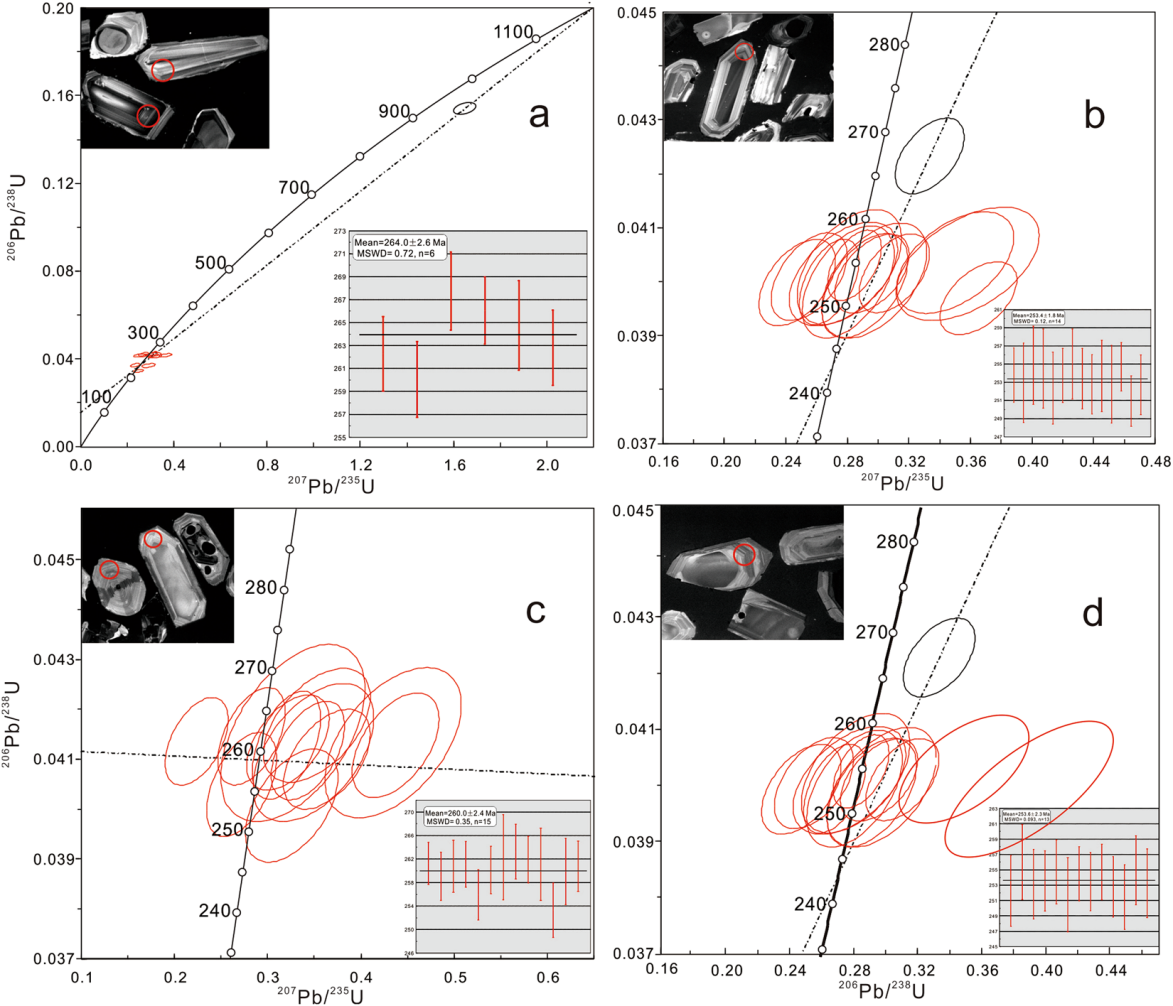
U-Pb Concordia diagrams for (**a**) CH-1, (**b**) CH-2, (**c**) SS-1, (**d**) SS-2.

The cathode luminescence (CL) images indicate that the dominant zircons are euhedral to sub-euhedral accompanied by prismatic, especially long prismatic shapes. The internal structures of the zircons are homogenous, oscillatory-zoned, or the oscillatory-zoned with homogeneous core in the volcanic ashes, suggesting their magmatic origin^16,33,34^. The weighted mean ^206^Pb/^238^U ages of the four samples are 264.0 ± 2.6 (CH-1), 253.4 ± 1.8 (CH-2), 260.0 ± 2.4 (SS-1) and 253.6 ± 2.3 Ma (SS-2), respectively (Fig. 2). For purpose of discussion, the zircon ages are interpreted to be inherited from a magmatic source and these represent the crystallization age of the original materials.

The ages of CH-2 and SS-2 are almost equal, which is near the PTB age. It is similar with the evidence of the sporopollen and conodont biostratigraphy which helps to confirm the PTB position. The ash beds of CH-1 and SS-1 also occur in the same time period within the error range of measurement.

### Mineral composition of the tuff samples

**Figure 3:**
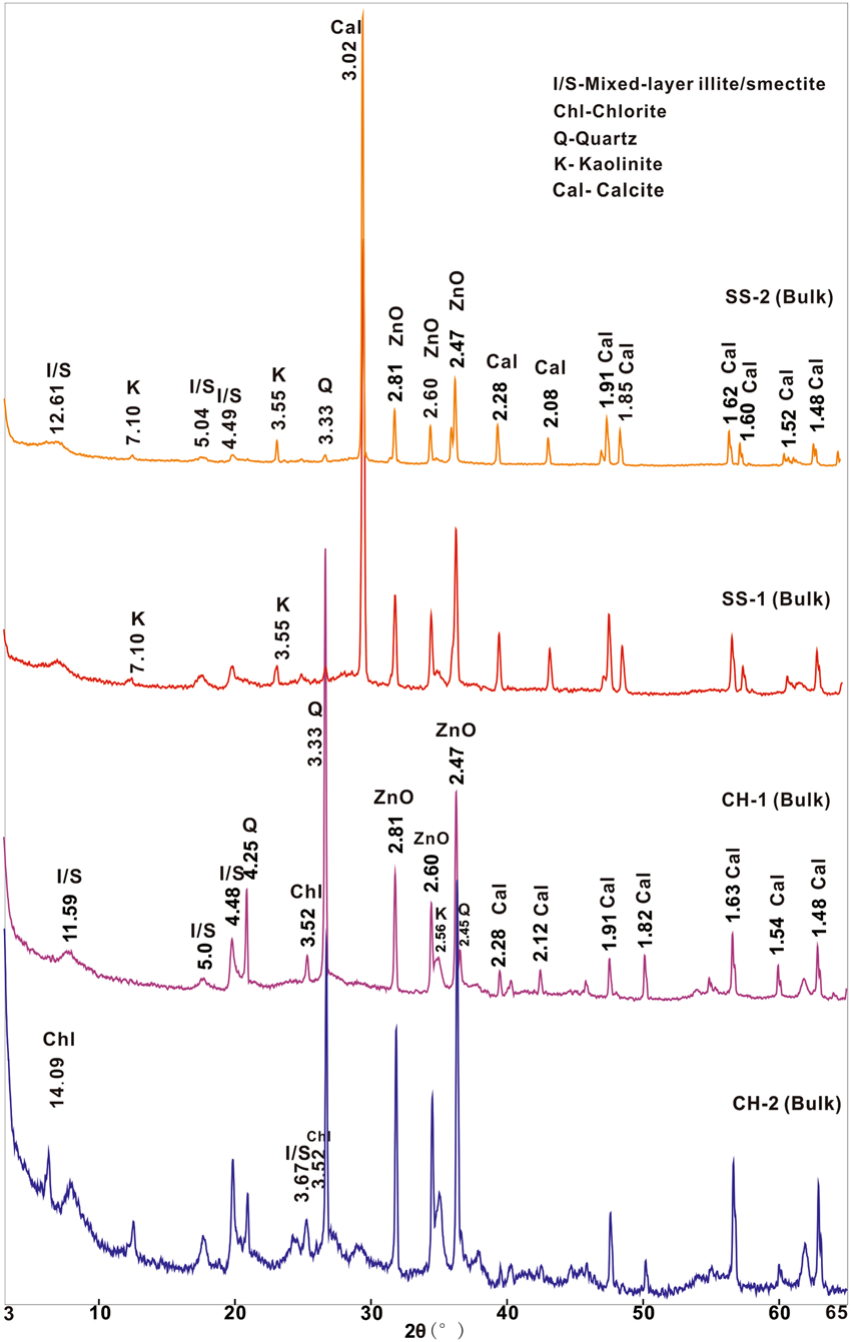
XRD profiles from bulk samples of Chahe and Shangsi altered ash samples.

The XRD results of bulk rocks are shown in Fig. 3. The results show that all the volcanic ash beds are dominated by illite-smectite (I-S) clay minerals. The minor minerals of the two sections show different compositions. CH-1 and CH-2 contain quartz and chlorite. And both of SS-1 and SS-2 are detected calcite, kaolinite and trace amounts of quartz.

### Clay mineral composition

**Figure 4:**
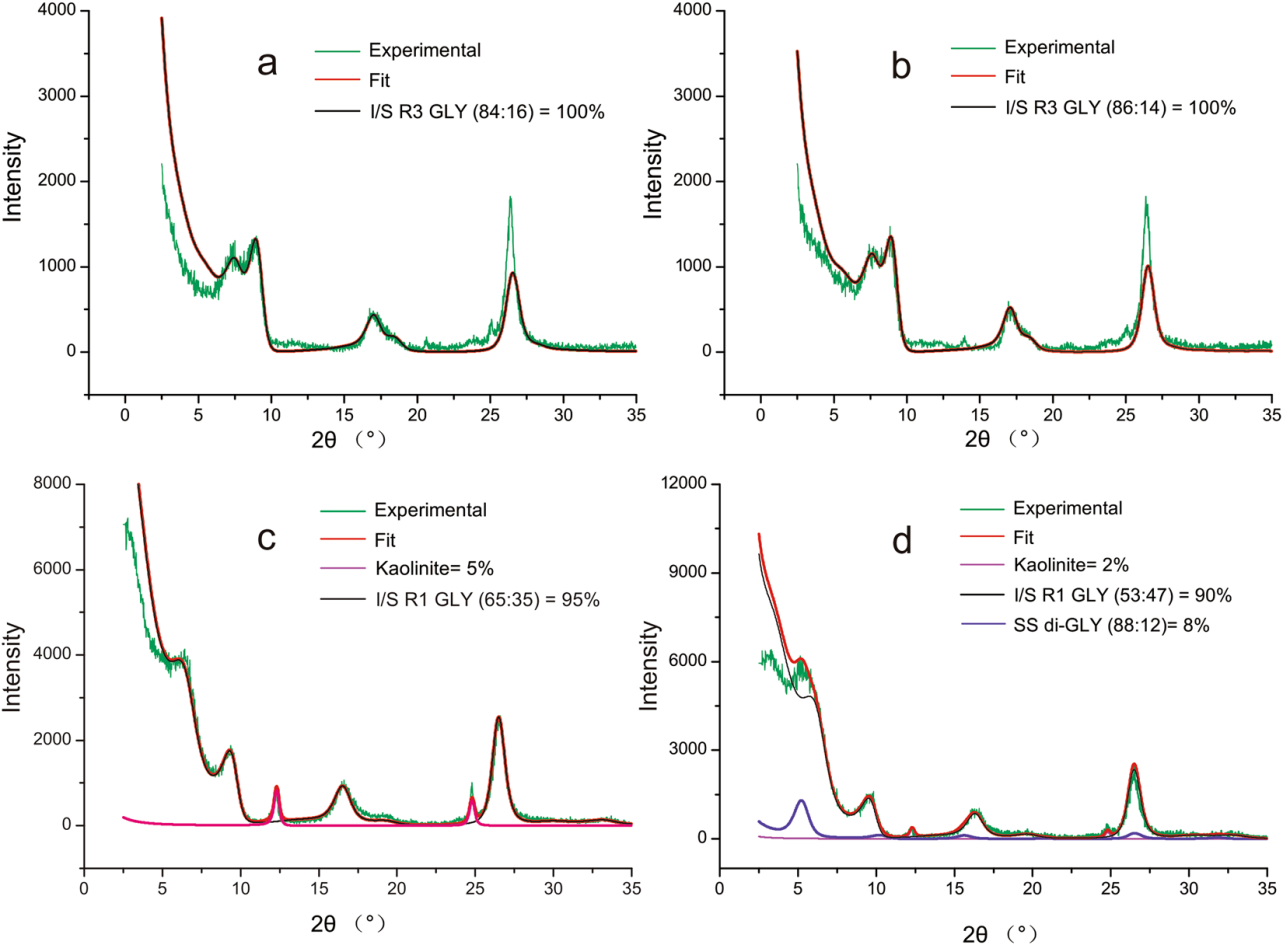
XRD profiles from Air-dried (AD) and EG-saturated (EG) of the I/S (illite-smectite) mixed layered clay minerals. The numerical values are the d values (nm) of the experimental patterns.

XRD spectra of the air-dried and EG clay fractions show that clay fractions from different depositional faces consist dominantly of mixed-layer illite-smectite (90–100%) (Fig. 4). I-S is identified by a relatively broad (001) peak between 10–12 Å in the air-dried fraction, and after EG solvation, it separated into two peaks at 11 Å and ~9.5 Å. Both of the CH samples display a strong peak at 11.0 Å, and separate into two peaks at 12 Å and ~10 Å after EG solvation (Fig. 4). The SS samples show a strong peak ~12 Å, and split into ~13 Å and ~9 Å after EG solvation (Fig. 4). Besides, the peaks of I-S are sharper and more intense in the case of CH, showing a more ordered structure. Except for the dominant I-S minerals, the SS clay samples contain trace amounts of kaolinite (~6%), while those of CH present with minor amounts of chlorite (7.04~, 3.52 and 14 Å) (<1%).

**Figure 5:**
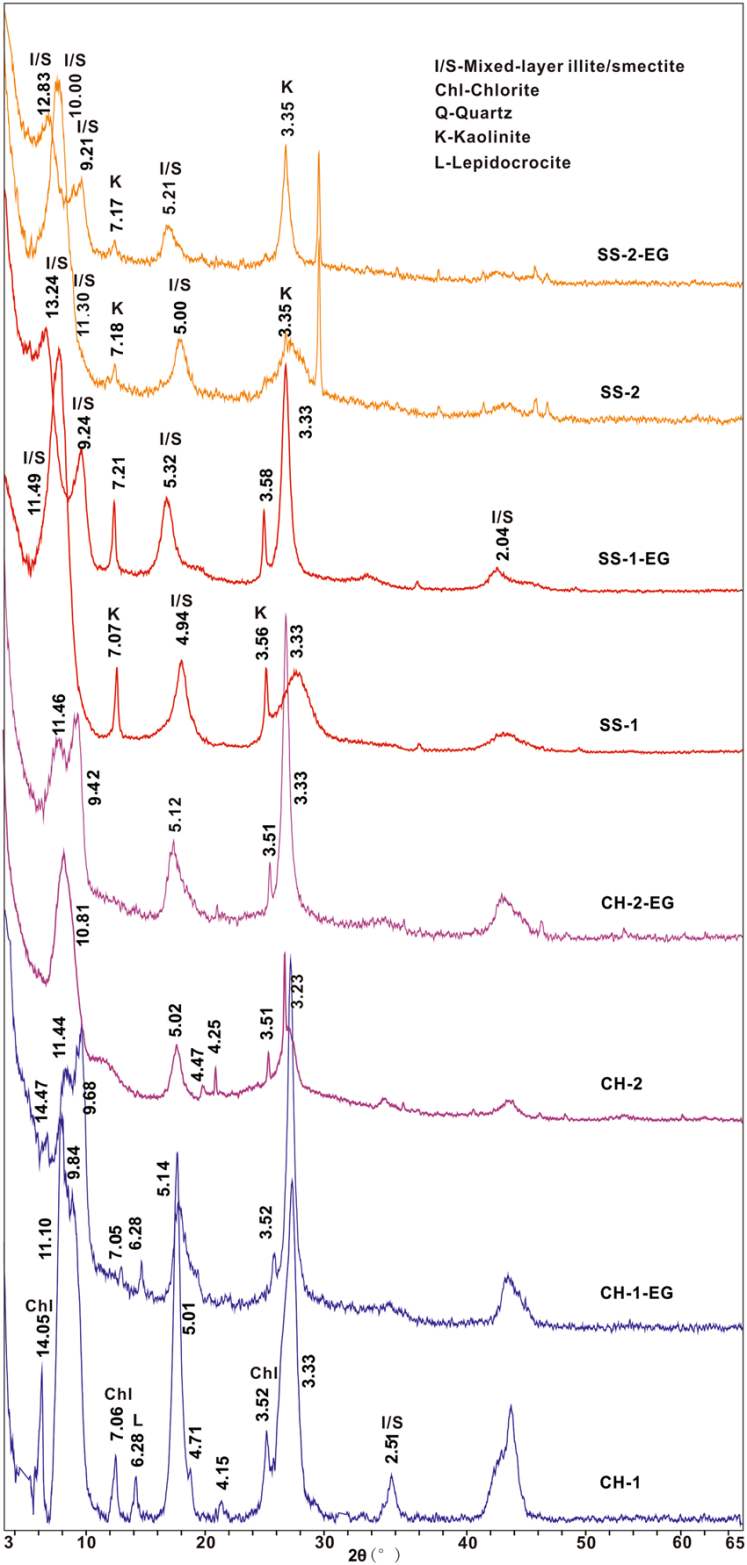
Experimental and calculated XRD patterns of the oriented, air-dry specimens from studied clay mineral samples ((a) CH-1, (b) CH-2, (c) SS-1, (d) SS-2).

The determination of I-S species was carried out by the NEWMOD program simulation of the XRD patterns^35^. The NEWMOD program generally produces a steeper background (calculated pattern) than the experimental pattern at the low-angle region^36^, as is also true for our samples. The accuracy of the fitting procedure was displayed by the content accordance between the experimental and calculated patterns. The simulation result shows that the I-S minerals of CH are more ordered than SS’ samples. The order of the I-S is also consistent with the percent of illite (I%): the layer structural order is R3 ordered for CH-1 (I% = 86%) and CH-2 (I% = 84%), R0 and R1 ordered for SS-1 (I% = 58%), and R1ordered for SS-2 (I% = 68%), where R is the Reichweite parameter (Fig. 5).

### Elemental geochemistry

The major-element contents of the four selected bulk samples are listed in Table 1. The Loss on ignition (LOI) value of tuffs of CH-1 and CH-2 are 9.42 wt% and 9.17 wt%, while the SS-1 and SS-2 are 31.28 wt% and 24.56 wt%. SS-1 and SS-2 show a higher content of Ca, Mg, but lower in Si, Fe, K, and Al.

**Table 1:** Major chemical compositions of the ash beds (wt%).

Sample	SiO_2_	TiO_2_	Al_2_O_3_	Fe_2_O_3_	MnO	MgO	CaO	Na_2_O	K_2_O	P_2_O_5_	LOI	CIA	SiO_2_/Al_2_O_3_	K_2_O/Al_2_O_3_
CH-1	57.47	2.62	21.61	3.73	0	0.79	0.31	0	4.45	0.05	9.42	82.05	2.66	0.21
CH-2	48.74	0.71	26.43	7.54	0.24	1.31	0.27	0.02	5.3	0.11	9.17	82.27	1.84	0.20
SS-1	31.26	0.31	14.15	1.96	0.02	1.68	23.43	0	2.16	0.06	24.56	86.04	2.21	0.15
SS-2	40.86	0.32	16.83	2.56	0.07	2.41	14.55	0.09	3.01	0.06	18.98	82.85	2.43	0.18

**Figure 6:**
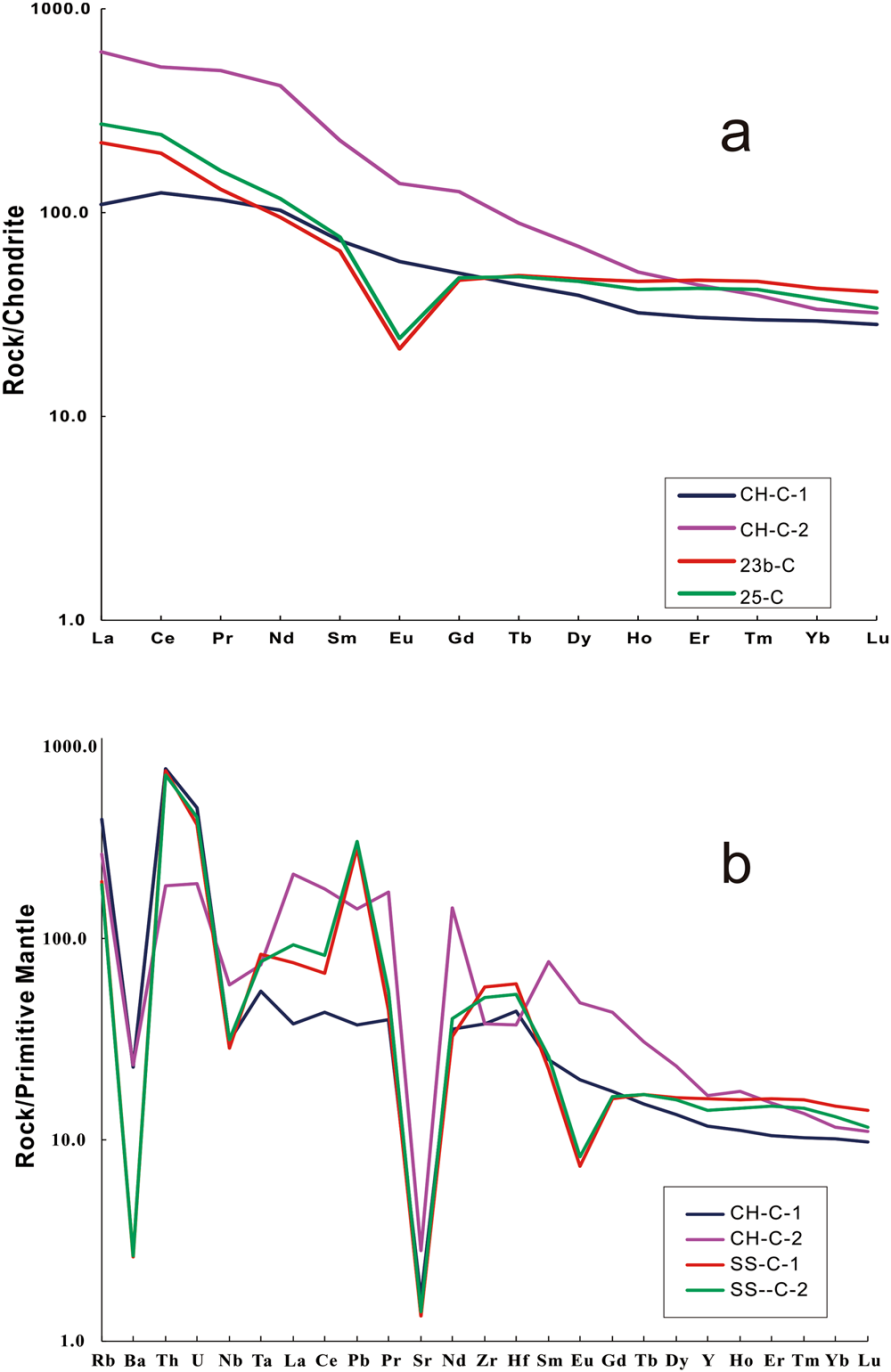
(**a**) Chondrite-normalized REE patterns. (**b**) Primitive mantle-normalized trace element patterns of the PTB volcanic ashes in South China. Chondrite and PM normalizing values from Sum and McDonough^37^.

**Figure 7:**
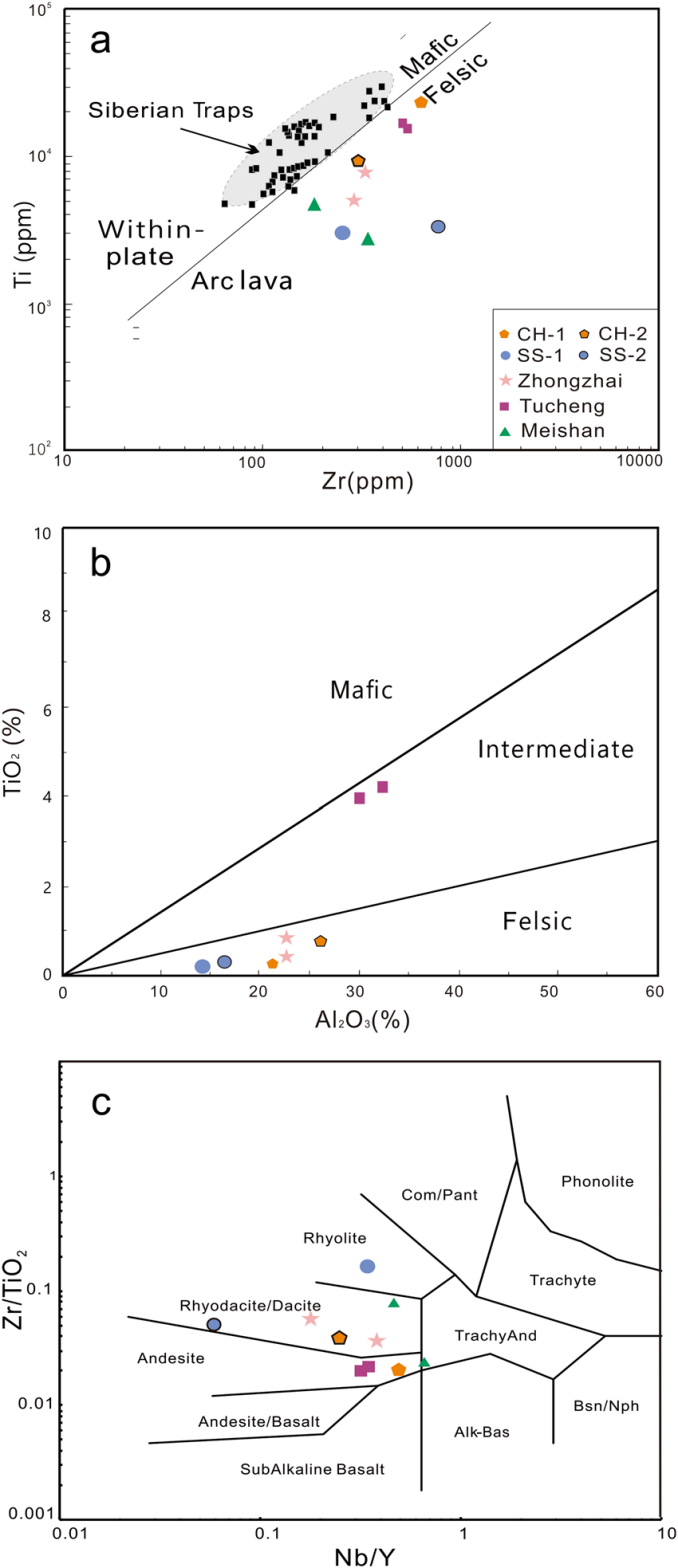
(**a**) Zr versus Ti diagram (after Pearce (1982)). (**b**) Plot of TiO_2_ and Al_2_O_3_. (3) Zr/TiO_2_ versus Nb/Y diagram.

Results of the trace-element analyses of the two sections’ clay minerals are presented in Table 2. Chondrite-normalized REE patterns^37^ of the investigated samples are shown in Fig. 6. The normalization of the study samples shows a generally large REE fraction and sloping curves with an integrally enrichment ratio of LREE (light rare earth elements) and depletion of HREE (heavy rare earth elements). The the clay minerals of CH-1 and CH-2 present slightly negative Eu anomalies (Eu/Eu* = 0.9493 and 0.8263), whereas the SS-1 and SS-2 show strongly negative Eu anomalies (Eu/Eu* = 0.4015 and 0.4596). Besides, the marine samples are relatively weakly enriched in HFSE (high field strength elements), and Zr and Hf show a slightly positive anomaly. ΣREE concentration is especially higher in CH-2 (805 ppm, which is 2.4–3.8 times higher than other samples (from 207 ppm to 334 ppm). The ΣLREE/ΣHREE ratios of terrestrial and marine sections are 5.02 (CH-1), 11.6 (CH-2) and 7.2 (SS-1), 9.2 (SS-2), respectively. Given the mobility of major elements during the argillization of the parent rock, the chemical discrimination diagram of the studied tuffs was used by Winchester & Floyd (1977), based on the concentrations of immobile elements, to differentiate the origin of the parent rock (Fig. 7).

**Table 2:** Tace elements data of the volcanic ashes (10^−6^).

Sample	Li	Be	Sc	V	Cr	Co	Ni	Cu	Zn	Ga	Rb	Sr	Y	Zr	Nb	Sn	Cs	Ba	La	Ce	Pr
CH-1	3.14	3.36	15.8	33.2	13.0	14.7	25.6	128	45.8	34.9	226	22.6	59.0	554	31.8	10.7	11.8	163	31.3	86.4	12.4
CH-2	1.62	5.08	32.6	265	214	5.25	28.2	132	27.2	28.8	105	35.5	132	295	31.9	3.00	3.01	108	222	341	84.4
SS-1	50.8	3.45	10.7	9.43	4.48	4.76	12.0	21.1	70.6	24.7	79.1	174	90.3	276	23.5	11.4	10.3	85.0	72.8	154	17.9
SS-2	53.7	2.69	10.7	25.4	5.31	10.6	27.2	16.6	69.3	26.2	107	160	66.3	536	26.5	9.50	12.0	45.3	78.2	168	19.3
Sample	Nd	Sm	Eu	Gd	Tb	Dy	Ho	Er	Tm	Yb	Lu	Hf	Ta	Tl	Pb	Th	U	δEu	ΣREE	ΣLREE/ΣHREE	
CH-1	53.2	12.2	3.88	11.7	1.90	11.0	2.11	5.74	0.86	5.67	0.81	17.0	2.97	0.54	2.58	69.9	10.9	0.9493	210	5.02	
CH-2	361	76.7	19.9	63.3	7.85	34.9	5.32	11.6	1.42	7.52	1.04	7.93	2.01	0.31	6.28	11.2	3.10	0.8263	805	11.6	
SS-1	62.8	13.8	0.91	12.7	2.41	15.3	3.20	9.97	1.54	9.51	1.35	9.21	2.59	0.36	44.9	43.8	9.60	0.4015	333	5.6	
SS-2	68.6	14.2	1.63	12.4	2.16	12.6	2.48	7.22	1.01	5.91	0.83	15.2	2.59	0.41	35.5	49.7	9.72	0.4596	207	7.2	

Note: δEu = Eu/Eu* = Eu_CN_/(Sm_CN_ × Gd_CN_)^1/2^, the subscript CN denotes chondrite-normalized, normalization values are after Sun and McDonough^37^.

### Sr-Nd and δ^18^O isotopic compositions

Sr and Nd isotopic concentrations of the clay mineral fractions along with the Rb/Sr ratios are listed in Table 3. For the studied samples, their initial ratio of ^87^Sr/^86^Sr and ε_Nd_(t) values are calculated at t = 250 Ma. All of the ash beds show a large variation of ^87^Sr/^86^Sr values ranging from 0.717225 to 0.761077. ^143^Nd/^144^Nd values are in the range between 0.512034 and 0.512424, and the ratios of CH section are relatively higher than those of SS. On the whole, The Rb/Sr ratio of CH-1 is higher than the SS samples, and the Rb/Sr ratio of CH-2 is notably lower than any other beds. As to oxygen isotope composition, the SS samples have much higher δ^18^O values (20.5‰ and 21.6‰) than those of CH section (13.2‰ and 13.4‰) (Table 3), and the δ^18^O values of the same sedimentary environments show little difference.

**Table 3:** Sr and Nd isotopic data and oxygen isotope values.

Sample	^87^Sr/^86^Sr	^143^Nd/^144^Nd	Rb/Sr	δ^18^O
CH-1	0.761077	0.512376	7.2	13.2
CH-2	0.742332	0.512424	2.8	13.4
SS-1	0.721708	0.512034	4.0	20.5
SS-2	0.717225	0.512043	5.1	21.6

## Discussion

### General petrology and geochemistry of the tuff

Tuffs are the altered remains of pyroclastic deposits. The original magma chemistry information can be principally obtained from immobile trace elements. All the beds have great ranges of Al_2_O_3_/TiO_2_ ratio from 8.24 to 52.59. TiO_2_, the HFSE (Nb, Ta, Zr, Hf), and REEs which are generally considered as immobile elements during diagenesis and weathering, are important indices for magmatic origin^14,20^. The Zr/TiO_2_ and Nb/Y ratios are commonly used as indicators of differentiation and alkalinity. Explosively erupted ashes tend to be characterized by high Nb content indicative of their high, silicic volatile feature^38^. As Al and Ti are immobile chemical components in the chemical weathering process^39^, their contents will remain constant in materials with different weathering degrees^40^. Thus, the Al_2_O_3_/TiO_2_ ratio has been proven to be a reliable proxy to indicate the provenance^41^. The nature of volcanic ashes of the study samples are classified as felsic and intermediate based on Al_2_O_3_ versus TiO_2_ diagram (Fig. 7). This is also confirmed by a cross plot of Nb/Y versus Zr/TiO_2_ (Fig. 7). The plot suggests that CH-1 and CH-2 are derived from andesite and rhyodacite, respectively, and SS-1 and SS-2 are from a rhyolite source.

For the Nd isotopic composition of the tuffs, the chemical weathering process exerts a negligible influence in terms of its primal parent rock undergoing a supergene environment. The ^143^Nd/^144^Nd ratio (Table 3) is another useful and sensitive indicator for the source rock. The ^143^Nd/^144^Nd ratio shows a difference between CH-1 and CH-2 (0.512376 and 0.512424), and a slight difference between SS-1 and SS-2 (0.512034 and 0.512043). Also, the CH section presents a significantly higher^143^Nd/^144^Nd value than that of the SS section. Significantly different Nb isotopic compositions, together with the Nb/Y-Zr/TiO_2_ discrimination diagram (Fig. 7), suggesting that the tuffs were derived from different magma sources for the beds of the CH and SS sections. The ash beds in the SS section present slightly different values of ^143^Nd/^144^Nd and fall into the field of rhyolite indicating that the volcanic ashes mostly likely have been derived from the same local volcanism and source rocks.

The isotopic composition of Sr of the marine section samples depends primarily on two major sources: the Sr isotope signatures in the oceans and the parent rock of the tuffs. Brachiopods and conodonts are identified as the most reliable material for measuring Sr isotope ratios in the oceans. The ^87^Sr/^86^Sr ratio was peaked in the PTB interval from late Permian to early Triassic^42,43^. The Sr isotope composition of conodont of Guandao section located in Guizhou is a plateau with minor fluctuations around 0.70710^44^, which is far below the Sr value of the SS tuffs (0.721708, and 0.717225). Therefore, the source of the rock played a dominant role on the high ^87^Sr/^86^Sr ratio of the samples in this study. The high ^87^Sr/^86^Sr ratio of voluminous silicic rocks is also mostly associated with ignimbrite flare-up eruptions and exhibits a high value, corresponding to available involvement of continental crust in arc magmatism^45^.

### Evaluation of diagenesis in marine and terrestrial sections

**Figure 8:**
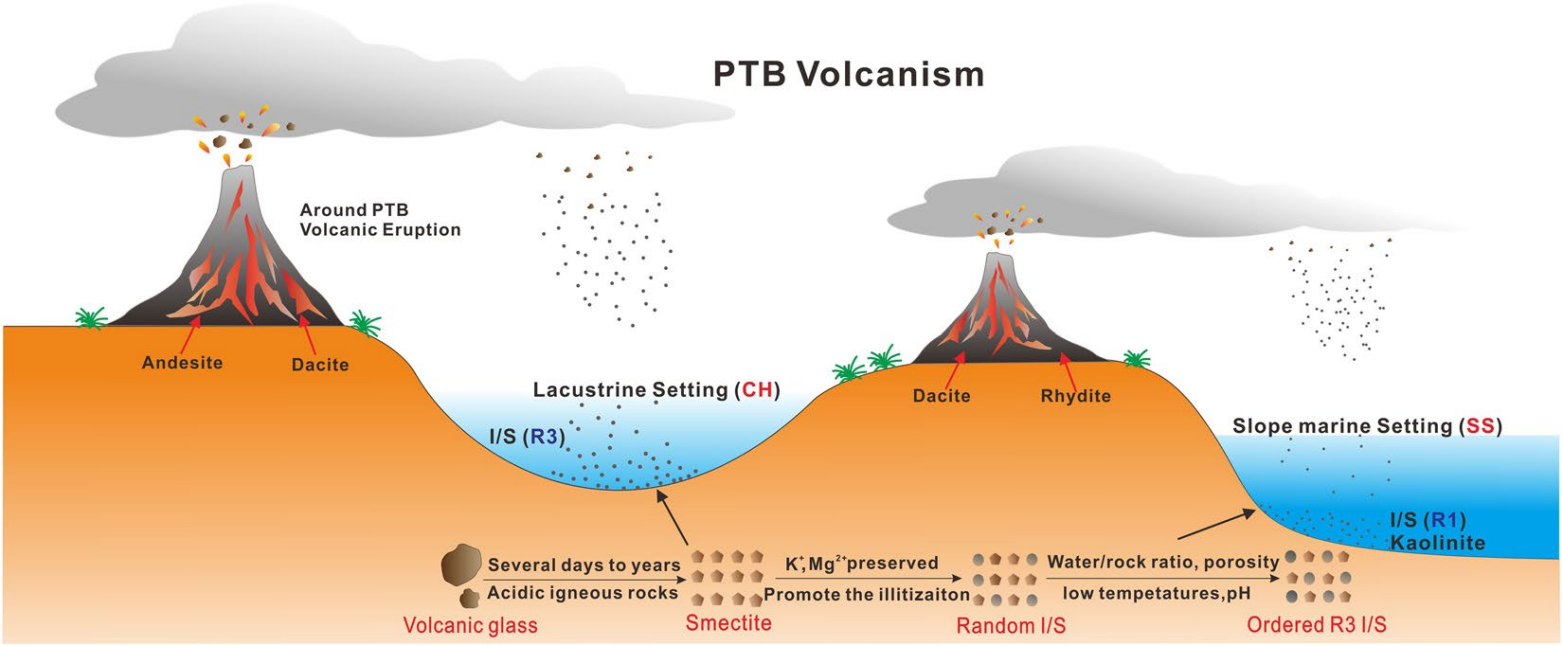
The evolution from the volcanic ashes to the I-S layer mineral in marine and terrestrial sedimentary environment in PTB.

The changes in the bulk sample composition of tuffs from different sedimentary facies are controlled by parent-rock composition, pH, organic matter, pore-water, and microbial activity^18,21^. Clay-mineral compositions of the samples are essentially I-S, with only just other minor clay minerals showing a few differences. The pyroclastic materials deposited in a marine environment were primarily altered into an association of K-feldspar and I-S^23^. The SS section samples show a trace amount of kaolinite. However, it is almost not detected in CH, while a minute amount of chlorite is detected in CH section (Fig. 8). Kaolinite and chlorite are the sensitive environmental indicators denoting different factors during its deposition and initial transformation. The minor detrital minerals of chlorite suggest relatively lower speed deposition of volcanic ashes^46^ in CH section. The kaolinite formation is typically formed from weathering, altered acidic igneous and metamorphic rocks or detrital products^47,48^. The presence of kaolinte suggests that it is from acidic igneous source rocks and its formation environment is reducing pH and well-drained leading conditions during alteration processes. The stability of clay minerals is inclined to increase along with the decreasing pH^23^. Because, the Al-OH species depends mainly on pH value, and aluminum cations tend to be produced under condition of pH ≤ 6^49^. Si is released gradually by smectite at relatively low pH which further promotes the transformation from smectite to illite. The slightly negative Eu in CH and strongly negative Eu in SS (Fig. 6) indicate that the lower pH of crustal environment induces the host-rock towards clay-dominated products. The strongly negative Eu anomaly of the SS may be inherited from the reduced environmental conditions during the formation of I-S, Eu^3+^, therefore, converted to Eu^2+^ and retained. The source of K in illitization can be provided from acidic magmas or high pH seawater^50^. Obviously the former can be considered as the main source of K to the transformation processes from smectite to illite.

In different sedimentary environments, volcanic glass in tuff alters into various minerals, such as the assemblages of the clay minerals, the type of mix-layer clay minerals and the REEs. Smectite converts to I-S at relatively slow and long-lasting time and low temperatures^51^. Illitic clays in volcanic ashes commonly replace the smectite precursor and inherit the texture with low porosity and permeability^52,53^. Different degrees of I-S interstratification in the smectite-to-illite transformation are controlled by primary rock lithology or the later sedimentary environment^20,54,55^, as identified by water/rock ratios, fluid chemistry and temperature. Evidence that the CH and SS beds are altered from different primary rocks is supported by the Nb/Y vs Zr/TiO_2_ diagram (Fig. 7) and the content of Nb (Table 3). Clearly, it is indicated that the sedimentary environments mainly influence the order and content of illitization, and the variable types of authigenic illite in the I-S mixed layer attribute to variations in porosity and permeability of the host rock^56,57^.

The degree of I-S and the content of illite in CH (R3) are higher than that of SS (R1) which have close relations with the sedimentary environment. The δ^18^O value of clay minerals is inherited from the water in the sedimentary environment, while the parent rock has little impact on the oxygen isotopic composition of altered clay minerals^58^. Water/rock ratios show an inverse relationship with the δ^18^O value, and the depletion of the oxygen isotope composition of the clays suggests a relatively high water/rock ratio related to fluid flow^59^. The influence of water/rock ratios occurs when the volcanic ashes are not greatly compacted during deep burial, and the lower water/rock ratio should theoretically correspond to a weaker leaching condition^56^. The SS I-S minerals have a much higher δ^18^O than that of CH I-S (Table 3), indicating a relatively low water/rock ratio. This is also consistent with the conventional wisdom that higher illitic I-S minerals precipitate at higher water/rock ratios^60^. In addition, the Fe content of the CH section is higher than that of SS integrally, also suggesting a deeper degree of illitization^61^. This reaction driven by Fe reduction. J.M. Huggett *et al*. (2005) suggest that Fe^2+^ promote the illite content.

There is a gradual increase in K_2_O and Al_2_O_3_ with a corresponding decrease in SiO_2_ and CaO, so the K_2_O/Al_2_O_3_ ratio (Table 1) can be used as a proxy linked for chemical weathering with Ca and Na removal. The K_2_O/Al_2_O_3_ value indicates the loss of K in mineral species during the weathering process, and therefore the relatively higher K_2_O/ Al_2_O_3_ ratio of CH than that of SS indicates a higher diagenetic grade and more intensely chemical weathering and alteration.

Unlike other homogeneous Nd isotopic compositions of I-S, the Sr isotopic compositions are more variable. The Sr isotope ratio can also be used to indicate the degree of chemical weathering which is proportional to the ^87^Sr/^86^Sr ratio^62^. Chemical weathering and alteration give rise to the leaching of Sr and increasing of the Rb/Sr ratio, and thus the ^87^Sr/^86^Sr ratio rises accordingly. The ^87^Sr/^86^Sr ratios (Table 3) of CH (0.761077 and 0.742332) section are obviously higher than those of the SS (0.721708 and 0.717225) section, consisting with the resulting formation of a higher degree of I-S and the content of illite in CH.

### Different sources of volcanism during the PTB period in south China

The nature of the volcanism about the formation of the P-T volcanic ash layers has been a controversial issue in south China. Previous studies proposed that the volcanic ashes may have been derived from the Emeishan basalt^63^ or the eruption of the Siberian Traps^27,64^. The convergent plate tectonics in the Panthalassa margin and Gondwanaland produced huge volumes of basaltic volcanism^4^, and intermediate-acidic volcanism^2,16^. Magmatic zircons exist widely in the tuff beds in south China. The hypothesis of the Siberian origin is questionable, because the Traps mainly consisted of mafic basalts, tuff in the bottommost and felsic at the upper part. Nevertheless, the PTB beds are primarily comprised of felsic tephras. Besides, the age of the Siberian Traps (251.7 Ma)^1^ is younger than the GSSP (252.28 Ma)^27^, and the PTB tuffs are correlated with crustal origin while the Siberian Traps is correlated with mantle origin. Thus, the causal relationships between the PTB volcanic ash and the Siberian Traps can be excluded. The thickness of the ash layers ranges from 13 cm to 30 cm in CH and SS, which is the same as other sections in Guizhou, such as the Xinmin, Duanshan and Zhejue sections. If the ashes were transported the long distances from the tectonic regions proposed above, this may not have resulted in ash layer thicknesses to this degree. The CH and SS section are situated closely in Lower Yangtze Regions, and the tuffs of the corresponding layers were formed contemporarily. The parent rocks of the studied tuff beds fall into the rhyodacite-andesite and rhyolite field separately whereas Meishan section located in the Middle-Upper Yangtze and falls into the rhyodacite-dacite field, indicating somewhat diverse source rocks. According to the studied sections, it can be assumed that the P–T transformation volcanism had a separated and regional magmatic source, not generated generally and identically. The volcanism in south China appears to be heterogeneous and not associated with a predominant and unitary source but derived from local volcanism or adjacent volcanism^17,65^.

## Conclusions

1. The tuff beds of the CH section, CH-2 from rhyodacite and CH-1 from andesite indicate that altered ash layers can be derived from different parent rocks in the same section. The same parent rocks can come from different depositional environments as the source of CH-2 and SS-2 are both rhyodacite.
2. The tuff of heterogeneous parent rocks in marine and non-marine sections both altered into I-S clay minerals, suggesting that the depositional environment plays a critical factor for the trend of alteration. The degree of illitization and the ordering of I-S, moreover, depend on the sedimentary environment mainly including the pH, ambient temperature and the water/rock ratios. Correspondingly, the degree of illitization and the I-S ordering, combining REEs, Nd isotopic and ^18^O, can be used as proxies to reflect the paleo-sedimentary environment. The terrestrial (CH) section reflects a higher diagenetic grade and more intense chemical weathering and alteration than the marine (SS) section, and both sections indicate that the relatively low pH environment did not experience the hydrothermal process.
3. Based on the studied terrestrial and marine facies occurring in almost the same period, the volcanism during the PTB transition can be regarded as heterogeneous in south China according to the geochemical fingerprinting of CH and SS. Thus, it is assumed that horizontal strata correlation of the tuff cannot be a reliable role of the source volcanism.

## Analytical techniques

### Mineralogical compositions

The mineral compositions were conducted by X-ray powder diffraction (XRD). Bulk samples that were strictly avoided from the detrital contamination and weathered portion during sampling were collected from the sections mentioned above. The air-dried whole-rock samples were crushed and ground to powders with a mortar and pestle, and then the clay mineral fractions (<2 μm) were extracted from the powder samples after centrifugation in distilled water using the sedimentation method^66^. The oriented mounts were prepared carefully by smearing the clay suspension onto glass slides and air dried. Air-dried (AD) oriented glass slides were treated in a container with ethylene glycol at 65 °C for 8 h in an electric oven. Ethylene glycol-saturated (EG) samples were to determine the occurrence of smectite, and mixed-layer illite-smectites. ZnO is used as the internal standard to analyze the clay minerals quantitatively by XRD.

X-ray diffraction analyses were performed using a Rigaku D/MAX-IIIA diffractometer (35 kV, 35 mA,) with Ni-filtered Cu Kα radiation from 2–65°2θ at a scan rate of 4° 2θ/min and with 1° divergence slit, 1° anti-scatter slit and 0.3 mm receiving slit.

### Zircon U-Pb dating

To separate the zircon crystals from K-bentonites, we picked zircon crystals located in the samples by hand under a binocular microscope. The potential target sites of zircons were selected via cathode luminescence (CL) images. Zircon U-Pb ages is dated through an inductively coupled plasma source mass spectrometer (LA-ICP-MS, Agilent 7500a) at GPMR of China University of Geosciences. External standard zircon, GEMOC GJ-1 (608.5 ± 15 Ma)^67^ and 91500 (1062 ± 4 Ma)^68^ were repeated to rectify the instrumental error and figure the element fractionations of Pb, Th, and U. The Si in NIST 610 glass is conducted as the internal standard to calibrate trace element compositions of zircon particles against multiple-reference materials^69^. The correction of Pb element adopts the method proposed by Andersen^70^. Off-line calculations, zircon U-Pb dating, and time-drift corrections were performed by the ICPMSDataCal program^69,71^.

### XRF analysis

The major-element concentrations of bulk samples were analyzed by wavelength dispersive X-ray fluorescence (XRF). Samples were powdered to <200 mesh size in an agate mortar, diluted 1:5 with dilithium tetraborate and fused using Philips Perl’ X3 automatic bead machine at 1200 °C. Measurements were carried out using a SHIMADZU XRF-1800 sequential X-ray fluorescence spectrometer. A pre-ignition was conducted to confirm the loss on ignition (LOI) prior to major element analyses. The detection limit was about 1 wt% and precision is <1% for major elements.

### Trace element and REE analysis

Trace-element and rare-earth-element (REE) analyses of bulk samples were conducted using an Agilent 7500a inductively coupled plasma mass spectrometer (ICP-MS), adopting the techniques presented by Liu *et al*.^71^. The analytical accuracy is <4% for REEs and Y, and 5–10% for other elements.

### Sr and Nd isotopic composition analysis

The composition analysis of Sr and Nd isotopic was measured as a Triton Ti thermal ionization mass spectrometer. Rb, Sr, and trace elements were detected by Resin columns AG50w × 8 and Dowe × 50w × 8 with 2.5 N HCl from the powdered samples. Isotope ratios of ^87^Sr/^86^Sr and ^143^Nd/^144^Nd were standardized respectively based on ^86^Sr/^88^Sr = 0.1194 and ^146^Nd/^144^Nd = 0.7219. The calibration of the instrument was repeatedly tested by standards NBS987 and La Jolla Nd_2_O_3_. Average values of the isotope ratios of ^87^Sr/^86^Sr and ^143^Nd/^144^Nd were 0.71026 ± 5 (2 SD) and 0.511845 ± 10 (2 SD).
